# Geometrically defined environments direct cell division rate and subcellular YAP localization in single mouse embryonic stem cells

**DOI:** 10.1038/s41598-021-88336-y

**Published:** 2021-04-29

**Authors:** Sarah Bertels, Mona Jaggy, Benjamin Richter, Stephan Keppler, Kerstin Weber, Elisa Genthner, Andrea C. Fischer, Michael Thiel, Martin Wegener, Alexandra M. Greiner, Tatjana J. Autenrieth, Martin Bastmeyer

**Affiliations:** 1 Zoological Institute, Cell- and Neurobiology, Karlsruhe Institute of Technology, Fritz-Haber-Weg 4, 76131 Karlsruhe, Germany; 2 Institute of Functional Interfaces, Karlsruhe Institute of Technology, Hermann-von-Helmholtz-Platz 1, 76344 Eggenstein-Leopoldshafen, Germany; 3 Institute of Applied Physics, Karlsruhe Institute of Technology, Wolfgang-Gaede-Straße 1, 76131 Karlsruhe, Germany; 4 Nanoscribe GmbH, Hermann-von-Helmholtz-Platz 1, 76344 Eggenstein-Leopoldshafen, Germany; 5 Institute of Nanotechnology, Karlsruhe Institute of Technology, Hermann-von-Helmholtz-Platz 1, 76344 Eggenstein-Leopoldshafen, Germany; 6 3DMM2O – Cluster of Excellence (EXC-2082/1 – 390761711), Karlsruhe, Germany; 7 Karlsruhe Institute of Technology, Hermann-von-Helmholtz-Platz 1, 76344 Eggenstein-Leopoldshafen, Germany

**Keywords:** Cell biology, Cell division, Pluripotent stem cells

## Abstract

Mechanotransduction via yes-associated protein (YAP) is a central mechanism for decision-making in mouse embryonic stem cells (mESCs). Nuclear localization of YAP is tightly connected to pluripotency and increases the cell division rate (CDR). How the geometry of the extracellular environment influences mechanotransduction, thereby YAP localization, and decision-making of single isolated mESCs is largely unknown. To investigate this relation, we produced well-defined 2D and 2.5D microenvironments and monitored CDR and subcellular YAP localization in single mESCs hence excluding cell–cell interactions. By systematically varying size and shape of the 2D and 2.5D substrates we observed that the geometry of the growth environment affects the CDR. Whereas CDR increases with increasing adhesive area in 2D, CDR is highest in small 2.5D micro-wells. Here, mESCs attach to all four walls and exhibit a cross-shaped cell and nuclear morphology. This observation indicates that changes in cell shape are linked to a high CDR. Inhibition of actomyosin activity abrogate these effects. Correspondingly, nuclear YAP localization decreases in inhibitor treated cells, suggesting a relation between cell shape, intracellular forces, and cell division rate. The simplicity of our system guarantees high standardization and reproducibility for monitoring stem cell reactions and allows addressing a variety of fundamental biological questions on a single cell level.

## Introduction

First isolated in 1981^1,2^ and utilized for genetic manipulation of mouse strains^3–5^, mouse embryonic stem cells (mESCs) have evolved to an excellent model for studying early developmental processes. Understanding and controlling self-renewal and differentiation are the two major goals in this field. In recent years it was demonstrated that mechanical properties from the environment evoke intracellular signaling cascades and direct gene expression and ultimately cell behavior^6^. A protein that has been identified in this context is the mechanotransducer yes-associated protein (YAP)^7^. It is highly expressed in mESCs^8^ and its nuclear localization supports pluripotency^9^. Remarkably, mechanotransduction via YAP shuttling can occur within two hours^7^. As a co-activator of the transcription factor TEAD, it promotes proliferation^10,11^ and self-renewal by enhancing the expression of pluripotency marker proteins^9,12–14^. Although the latter is widely accepted, there are contradictory results concerning YAP subcellular localization in mESCs. On the one hand, it has been demonstrated that YAP is localized to the nucleus and the cytoplasm^9^. On the other hand, it was shown that within the inner cell mass (ICM) YAP is inactivated, shuttled to the cytoplasm, and excluded from the nucleus to prevent differentiation into trophectoderm^15^. As many other proteins, relevant for early embryogenesis, YAP seems to be distributed within the cell in a context-specific manner. To steer self-renewal and differentiation of mESCs in culture, it is beneficial to understand how the nuclear to cytoplasmic (n/c) YAP ratio is determined. However, whether and how the environment affects YAP localization and ESC fate decision has not been sufficiently assessed. Furthermore, it is now well accepted that the response of various cell types drastically differs between 2 and 3D growth environments^16–18^. Accordingly, different types of 3D growth substrates have been developed to cultivate and manipulate various cell types, including ESCs^19–21^. Most of these 3D environments are based on complex matrices^22,23^ and experiments with ESCs are mainly performed with ESC colonies^24,25^. Although frequently demanded, there is still a lack of studies on single ESC fate decision with respect to clearly defined environmental cues. This is due to the fact that reliably assigning a specific cue to a cellular reaction within a complex growth environment is difficult^26,27^. As a first requirement, a cell culture system allowing for single cell observation needs to be designed. The setup should only provide the elementary environmental cues in a highly defined manner. In this research article, we describe our interdisciplinary approach to develop a robust and effective cell culture system that allows studying ESC fate decision on a single cellular level. We used two-dimensional (2D) micro-islands and micro-wells with a squared base of different sizes surrounded by walls (2.5D) to achieve different geometrically defined growth substrates. The varying degrees of confinement direct mESC morphology, cellular tension and presumably fate decision. While most former studies have investigated the impact of biochemical factors on ESC lineage specification^28–31^, only little is known about the impact of biophysical factors from the microenvironment on early fate decision events of single mESCs. Therefore, we explicitly focused on single mESC behavior shortly after seeding. We analyzed two short-term events during mESC fate decision: (1) the cell division rate, which decreases upon differentiation^32,33^ and (2) the context-dependent subcellular localization of YAP. We monitored the cells either during decision-making at 4 h or at 24 h to evaluate the respective outcome. In our cell culture system with a reduced environmental complexity we discovered that cellular confinement directs cell shape which influences subcellular YAP localization and ultimately the cell division rate of single mESCs.

## Results

### 2D and 2.5D confinement differentially affects mESC division rate

**Figure 1:**
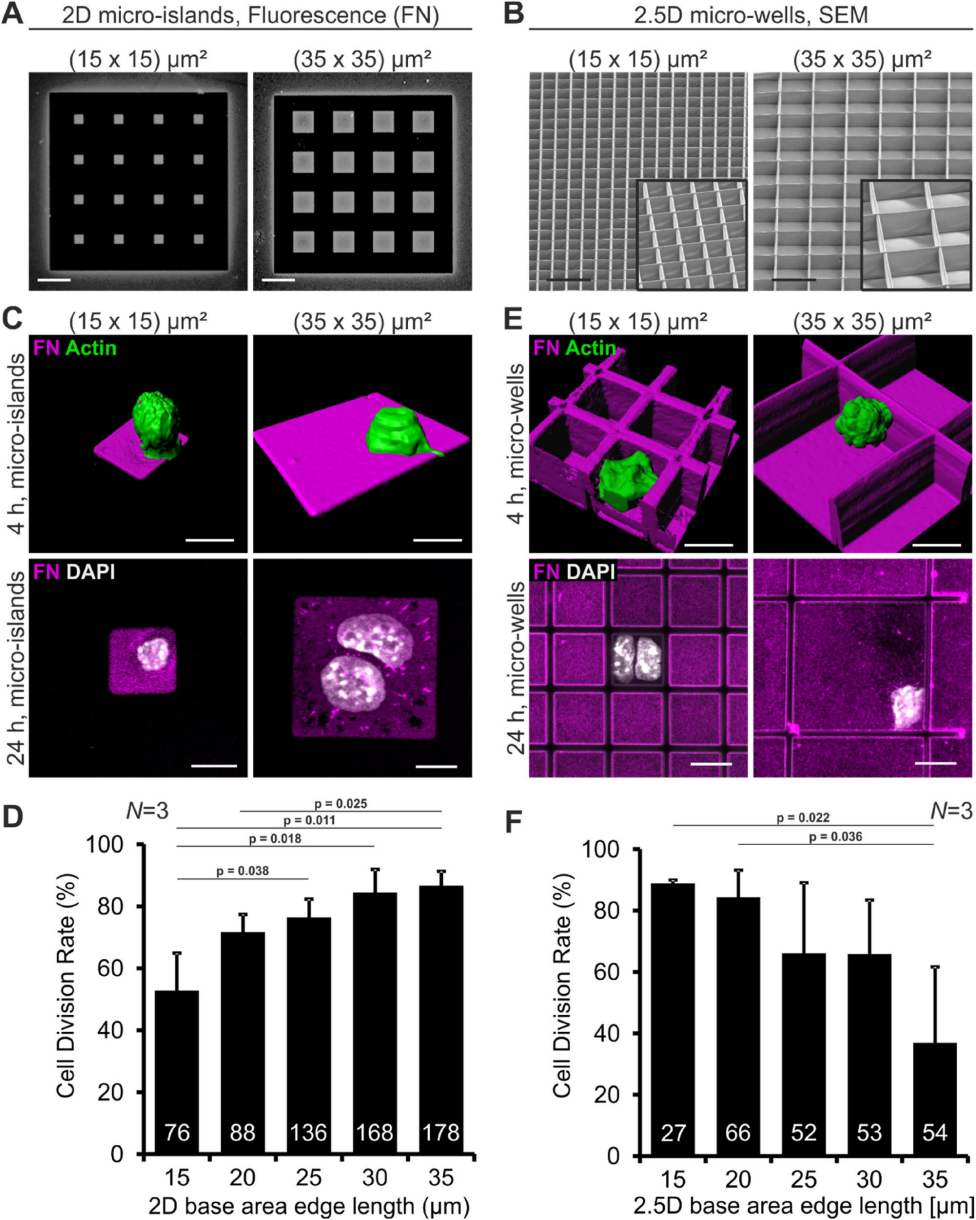
Cell division rate of mESCs is influenced by their 2D and 2.5D growth environment. (**A**) Fluorescence images of 2D micro-islands with a base area edge length of 15 and 35 µm, respectively. The micropattern was produced by microcontact printing. The islands were coated with fibronectin (FN, grey). The black areas are passivated and do not allow protein binding or cell adhesion. (**B**) Scanning electron micrographs (SEM) of the smallest ((15 × 15) µm^2^) and largest ((35 × 35) µm^2^) 2.5D micro-wells fabricated by direct laser writing. The walls have a height of approximately 20 µm. The insert shows a zoom-in at a different angle. Scale bars: 50 µm. (**C**) Representative images of mESCs on small and large 2D micro-islands. Upper row: 3D reconstructions from laser scanning image stacks of actin labeled (green) mESCs on FN (magenta) islands 4 h after seeding. Lower row: Images of mESCs 24 h after seeding. On the small island only one cell is present, whereas on the large island two nuclei (DAPI, white) are detected. (**D**) Quantification of the CDR of mESCs on micro-islands with different base areas. (**E**) Representative images of mESCs in small and large 2.5D micro-wells. Upper row: 3D reconstructions of single mESCs growing in FN (magenta)-coated micro-wells 4 h after seeding. In small micro-wells mESCs have a cross shaped morphology, in large micro-wells they display a round morphology. Lower row: Images of mESCs 24 h after seeding. In the small well two cells are present, whereas in the large well only one nucleus (DAPI, white) is detected. (**F**) Quantification of the CDR of mESCs in micro-wells. Scale bars: 10 µm. Graphs show mean ± one standard deviation (s.d.) from *N* = 3 independent experiments. The number of analyzed cells is given in the individual bars; *p* value was determined by two-tailed student’s t-test.

We first asked whether single mouse ESCs (mESC) can be influenced by the provided 2D and 2.5D adhesive area. 2D micro-islands were produced by microcontact printing, a soft lithography technique which enables patterning of surfaces with proteins separated by protein- and cell-repellent regions. 2.5D micro-wells were produced by direct laser writing (DLW), a technique which allows the fabrication of arbitrary free-standing structures in the range of single cells. Thus, DLW is a valuable method to produce well-defined 3D cell culture scaffolds^17^. The micro-islands and micro-wells were produced in different sizes – the edge length of the base area increased from 15, 20, 25, 30 to 35 µm in length, the height of the wells was kept at approximately 20–25 µm (Fig. 1A,B). We chose a minimal square size of (15 × 15) µm^2^ because mESCs in suspension have a diameter of 10–12 µm and cover the whole (15 × 15) µm^2^ micro-island already after four hours (Supplementary Fig. 1). Base areas and walls of the micro-wells were coated with the protein fibronectin (FN) to provide a minimal environmental cue that mimics the extracellular matrix (ECM). Single mESCs expressing the pluripotency reporter-construct OCT4-eGFP^34^ were cultured on the islands and inside the wells. Since the cell cycle of mESCs takes about 12 h^35^ we analyzed the cells 4 and 24 h after seeding to monitor initial division events. At the first time point, we documented the position of islands or wells containing only one single OCT4-eGFP-positive mESCs demonstrating the pluripotent state (Supplementary Fig. 2). In supplemental experiments, as a second pluripotency marker SOX2 was monitored by immunofluorescence (Supplementary Fig. 2). After 24 h the samples were fixed, stained and the same positions were imaged again. The images were compared and cell division events were counted. We found that on 2D substrates mainly mESCs on large islands underwent cell division (Fig. 1C). The quantification demonstrates that CDR depends on micro-island size. From small to large islands the CDR gradually increased from 53 to 87% (Fig. 1D). Surprisingly, mESCs in small 2.5D wells underwent cell division more often than mESCs in larger wells (Fig. 1E). From small to large wells the CDR gradually decreased from 89 to 37% (Fig. 1F). These findings were confirmed using EdU-incorporation assays (Supplementary Fig. 3). Thus, our findings demonstrate that the provided adhesive area as well as the dimensionality of the microenvironment affects cell division rate of mESCs. Notably, CDR correlates positively with adhesion area in 2D, whereas in 2.5D scaffolds CDR correlates with the degree of confinement.

### Strong 2.5D confinement provokes unique cellular and nuclear shape and maintains a high CDR

**Figure 2:**
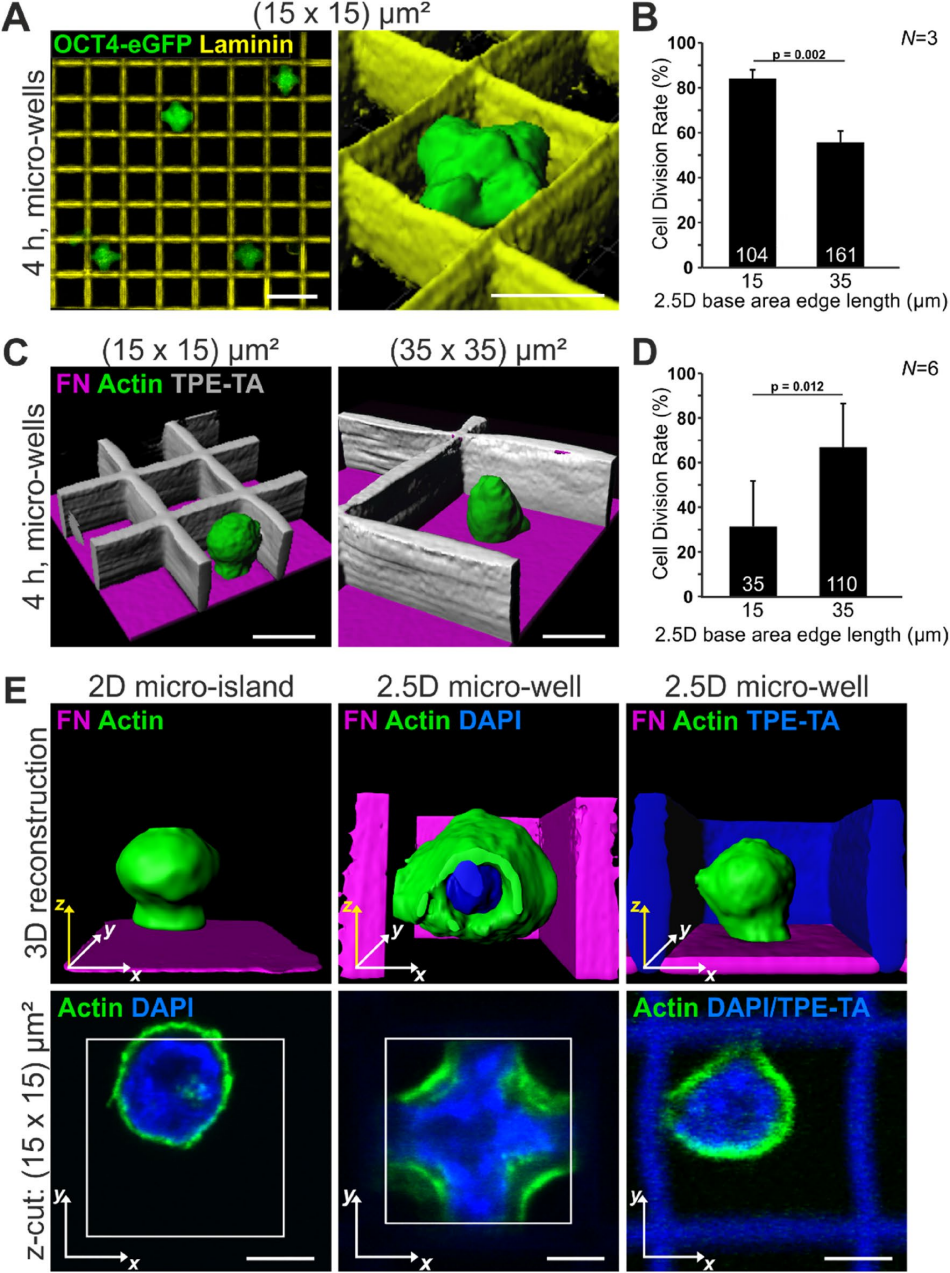
Cell shape is linked to cell division rate. (**A**) Single OCT4-eGFP expressing mESCs (green) in laminin-coated 2.5D micro-wells (LN, yellow) with 15 µm base area edge length four hours after seeding. The nuclei (marked by OCT4) exhibit a cross-shaped morphology. Scale bars: 10 µm and 15 µm for the 3D reconstruction. (**B**) CDR on LN significantly decreases with increasing micro-well size. (**C**) 3D reconstructions of mESCs in micro-wells with non-adhesive walls (TPE-TA, grey) and a FN-coated bottom (magenta). The cells exhibit a round morphology irrespective of the well size. Scale bars: 10 µm (**D**) The CDR increases with increasing micro-well size, as on 2D islands. (**E**) 3D reconstructions (upper row) and single optical sections (lower row) of mESCs four hours after seeding. Actin (green) and the nucleus (DAPI, blue) were stained. On 2D micro-islands and in 2.5D micro-wells with non-adhesive walls cells and nuclei have a round morphology. Cells (and nuclei) in 2.5D micro-wells with adhesive walls adopt a cross-shaped morphology with prominent actin arcs. Scale bars: 5 µm. Graphs show mean ± one s.d. from *N* = 3 independent experiments. The number of analyzed cells is given in the individual bars; *p* value was determined by two-tailed student’s t-test.

To carefully dissect what particular parameter promotes the high CDR in small micro-wells, we varied defined environmental cues within our system. To exclude that FN is responsible for the observed effects we changed the protein coating to laminin (LN) (Fig. 2A), another ECM protein of the early mouse embryo^36^. The behavior of single mESCs in LN-coated micro-wells did not differ from the behavior on FN. Again the CDR decreased from 84 to 56% with increasing micro-well size (Fig. 2B). Seemingly, the type of cell-ECM contact is not decisive for mESC behavior in our system. As an additional control, coating with Poly-d-Lysine (PDL) was performed. mESCs in PDL-coated scaffolds react in a similar manner with a higher CDR in small micro-wells (Supplementary Fig. 4). Another reason for the different cellular reactions could be the accumulation of factors secreted by the mESCs within the 2.5D micro-wells that possibly influence mESC fate decision. Therefore, we created a second setup with 2.5D micro-wells with non-adhesive walls, allowing potential accumulation of soluble factors (Fig. 2C). The walls of these scaffolds were fabricated from a photoresist with passivating properties (TPE-TA^37^), preventing cell adhesion. The bottom was the glass surface of the cover slips coated with FN. In these wells, the obtained CDR corresponded to the CDR on 2D where no accumulation of soluble factors can occur: The larger the base area, the higher the CDR of single mESCs (Fig. 2D). Thus, a potential accumulation of soluble factors within the micro-wells is not responsible for the observed effects. Additionally, we found that single mESCs cultivated in passivated 2.5D micro-wells did not interact with the walls of the scaffolds and resembled mESCs on 2D with respect to cellular morphology.

We next analyzed how many walls were contacted by the mESCs in FN-coated 2.5D micro-wells. We found that 80% of mESCs in small wells ((15 × 15) µm^2^) had contact to all four walls (as quantified in Supplementary Fig. 5), leading to cross-shaped cells (FN, Fig. 1E and LN, Fig. 2A). In large 2.5D wells ((35 × 35) µm^2^) most mESCs adhered to the bottom and in addition to one or two walls, leading to roundish cells (FN, Fig. 1E and LN, Fig. 2A). This is in contrast to cells growing on large 2D micro-islands, where the cells can easily spread as observed by live cell imaging (Supplementary Fig. 6). This led to the assumption that the type of cell adhesion and thereby cell shape is one relevant factor for decision-making in mESCs.

Accordingly, we compared mESC morphology on small micro-islands, in small micro-wells and in small micro-wells with non-adhesive walls. Single optical sections of mESCs reveal that only cultivation in small ECM-coated micro-wells led to the formation of prominent actin arcs and in addition resulted in a cross-shaped nucleus (Fig. 2E). In larger 2.5D micro-wells with adhesive walls the distance between two opposite walls prevents mESCs from spanning across the well. We quantified this finding and found that the cells in 2.5D micro-wells with a base area of (35 × 35) µm^2^ are mostly attached to one or two walls, do not spread, and reveal either a spherical or hemispherical morphology (Supplementary Fig. 5). On large 2D micro-islands, however, mESCs usually increase their spreading area before cell division (Supplementary Fig. 6). Cell spreading, as observed for mESCs in small micro-wells and on large micro-islands, is known to affect cellular tension. Thus our data suggest that an increased cellular tension correlates with a high CDR.

### Relative YAP localization in single confined mESCs correlates with cellular tension

**Figure 3:**
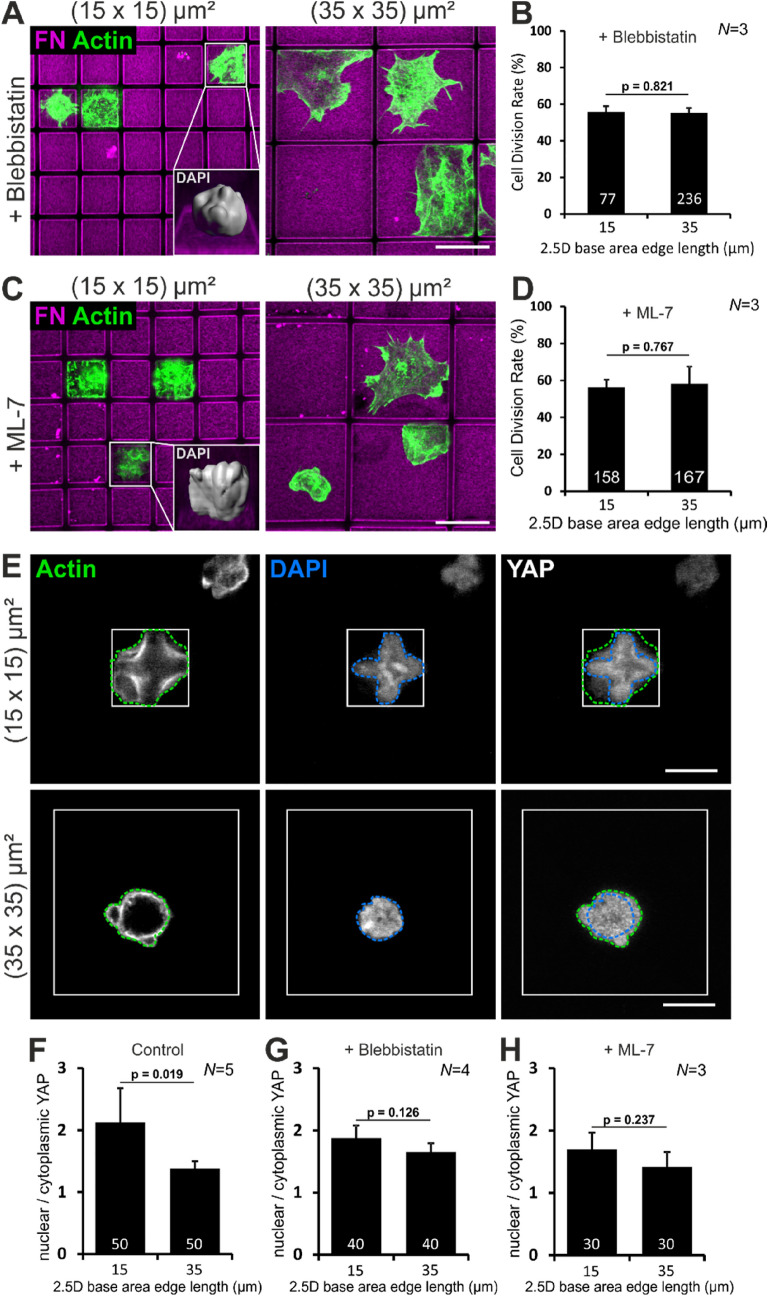
Changes in cellular tension lead to YAP activation. (**A**,**C**) Images of Blebbistatin or ML-7 treated mESCs in FN–coated micro-wells (magenta) with 15 and 35 µm base area edge length, respectively. Actin staining (green) demonstrates a large spreading area. Insets show 3D reconstruction of the nuclei of inhibitor treated cells in small micro-wells. Scale bars: 10 µm. (**B**,**D**) CDR of inhibitor treated mESCs does not differ between small and large micro-wells. The no-treatment control is shown in Fig. 1F. (**E**) Images of mESCs in small and large micro-wells four hours after seeding. The outline of the cell was determined by the actin signal (yellow line). The nuclear outline was determined by DAPI staining (blue line). Scale bars: 10 µm. (**F**) Quantification of the nuclear to cytoplasmic (n/c) YAP ratio in control cells. (G and H) Quantification of the n/c YAP ratio in inhibitor treated mESCs. Graphs show mean ± one s.d. from *N* = 5, 4, 3, respectively, independent experiments. The number of analyzed cells is given in the individual bars; *p* value was determined by two-tailed student’s t-test.

To confirm that cellular tension within single mESCs determines the CDR we applied two inhibitors that relieve actomyosin contractility. We applied the inhibitors at concentrations that affected cellular morphology but still allowed cell division. When imaged four hours after seeding, mESCs treated with the myosin II inhibitor Blebbistatin showed a flattened and enlarged phenotype compared to control cells. This effect was independent from micro-well size (Fig. 3A) and led to an equal CDR of ~ 55% in small and large micro-wells (Fig. 3B). In addition, loss of cellular tension also abolished the formation of cross-shaped nuclei in small micro-wells (Fig. 3A inset). The same observations were made for treatment with the myosin light chain kinase inhibitor ML-7. Again, the cells had a large spreading area (Fig. 3C) and an equal CDR of ~ 56% in small and large micro-wells (Fig. 3D).

It has been described that low nuclear stiffness, which is typical for mESCs, together with a high cellular strain due to actin stress fibers lead to a shift of mechanotransductive events from the cytoplasm to the nucleus^38^. This supports the notion that nuclear YAP is relevant for cross-shaped mESCs. We measured the relative nuclear to cytoplasmic localization of the mechanotransducer and transcriptional co-activator YAP.

The outline of the cell was defined by actin-labeling and the outline of the nucleus was determined by DAPI-staining. Subsequently, the mean fluorescence intensity of the YAP-signal was measured for the cytoplasm and the nucleus (Fig. 3E). Interestingly, our measurements revealed that mESCs in a confined 2.5D microenvironment in general have more nuclear than cytoplasmic YAP, thereby addressing the ongoing discussion on the relative YAP localization in mESCs.

The quantifications revealed that the nuclear to cytoplasmic (n/c) YAP ratio drops from 2.1 in confined, cross-shaped mESCs in small micro-wells to 1.4 in round mESCs in large micro-wells (Fig. 3F). The relative localization of YAP in inhibitor treated single mESCs further confirmed the assumption that the observed changes in CDR are dependent on cellular tension. For both inhibitors the n/c YAP ratio did not significantly differ between single mESCs in small or large micro-wells (Fig. 3G,H).

We conclude that for single mESCs in small micro-wells the confinement leads to a stretched phenotype, inducing high cellular tension that is translated into a biochemical signal via YAP translocation.

## Discussion

In recent years the influence of biochemical and biophysical factors from the local microenvironment on stem cell differentiation has frequently been studied. Most 3D cell culture systems, however, are too complex to determine the impact of specific environmental cues on stem cell fate decision. For example, gel-matrices, especially those derived from natural polymers such as collagen or hyaluronic acid, are difficult to standardize due to the random cross-linking process^39,40^. Additionally, characterization of the bulk material often fails to represent the local microenvironment which would be more relevant for cells^41^. Unfortunately, characterization of the local environment proved to be difficult and heterogeneity in local stiffness, pore size, permeability, ligand spacing or degradability has been observed^39,42^. Within our 2.5D cell culture system essential parameters, *i.e.*, adhesion area, dimensionality, and ECM-coating, are presented and systematically varied. Thereby, we can easily standardize the composition of our growth substrates which leads to highly reproducible results. Furthermore, we are able to monitor single mESCs instead of whole colonies. Protected from direct cell–cell interactions, the observed behavior can be assigned to the changes of the microenvironment. It was already proven for adult stem cells, especially mesenchymal stem cells that biophysical cues from the microenvironment regulate cell fate decision^43,44^. A typical cellular reaction of mESCs to the geometrical and/or mechanical properties of the microenvironment is the adaptation of their morphology. Interestingly, differences in cell shape have been shown to be closely linked to changes in cellular tension, both in 2D and in 3D^45,46^. As an example, intracellular tension caused by cell morphology is involved in the first specification event during mammalian embryonic development. At the 8-cell stage of mouse development an asymmetric cell division produces two daughter cells, one with weak and one with strong actomyosin contractility. Mediated through nuclear YAP, the highly contractile and stretched outer cells build the trophectoderm (TE) whereas the round and less contractile cells build the ICM^47^ which will form the embryonic tissue.

So far it has been shown that nuclear YAP positively correlates with cell proliferation^10,11^ and self-renewal of mESCs^9,12–14^. How dimensionality, cellular confinement, and mechanical forces influence the behavior of single ESCs is still a matter of investigation. To demonstrate the power of single cell monitoring in this context, we applied our geometrically defined growth substrates to analyze the CDR and subcellular localization of YAP with respect to different degrees of confinement.

In this study we determined the CDR by counting cell division events and not by measuring cell cycle length. Early in development, mESC have a doubling time of 10–14 h ^35,48^. Since we investigated isolated single mESCs we expected a prolonged cell cycle length. To be on the safe side, we therefore monitored the cells 24 h after seeding. For example, on the largest 2D micro-islands in 80% of the cases cell division events have been observed within these 24 h. Of these, 70% have divided only once, whereas the remaining 30% have undergone two rounds of division. This roughly corresponds to a cell cycle length of 15 h under our experimental conditions.

In this context we want to mention that we did not distinguish between single mESCs that correspond to the early (naïve) state of development or the late (primed) state of development.

Pluripotency is not a fixed state of embryonic stem cells before they undergo differentiation but rather a spectrum ranging from naïve to primed cells^49,50^. Naïve pluripotent cells have a larger developmental potential as primed cells. This becomes evident, e.g., during chimera formation, where only the naïve population contributes to chimeric embryos. Depending on the culture conditions different states of pluripotency are supported. In this study mESCs have been cultured in medium supplemented with LIF and serum. These cells represent a heterogeneous population ranging from the naïve pluripotent state to the primed pluripotent state. The mESCs thus exhibit a heterogeneous expression of different pluripotency markers^51,52^. OCT4, used in this study, marks all pluripotent cells irrespective of their state but is lost upon full commitment of the cells. Therefore, we cannot distinguish between naïve and primed mESCs but accordingly we included the whole spectrum of pluripotency into our analysis.

By systematically varying size and shape of the 2D and 2.5D substrates we observed that the geometry of the growth environment affects the CDR: Whereas CDR increases with increasing adhesive area in 2D, CDR is highest in small 2.5D micro-wells and decreases with increasing well size. One interpretation for these results could be that the CDR is proportional to the cell/ECM contact area. This could especially explain the situation on 2D substrates, where the cells could form more contact sites and thus increase the amount of intracellular signaling cascades that regulate cell division^53^. This contact is in general mediated by integrins that upon contact to ECM cluster and establish intracellular signaling complexes. Unfortunately, we have been unable to quantify cell/ECM contact area since the mESCs form mostly small and diffuse focal adhesions as monitored by Paxillin staining. This is not surprising since low focal adhesion signaling is a hallmark of mouse embryonic pluripotency ^54^. On the other hand, it has been shown for adult stem cells and progenitors that not only the amount of ECM but also the geometrical distribution can at least influence fate decision^43,44,55^. We find that mESCs on larger 2D islands spread before division indicating that these cells have an increased intracellular tension that could be correlated to CDR. Concerning mESCs in 2.5D substrates, we find that CDR is highest in small micro-wells. Here, the high CDR cannot be explained solely by the magnified cell/ECM contact area since the mESCs have a raised position and do not contact the ECM-coated bottom of the well. When these cells were treated with inhibitors of myosin contractility they are no longer elevated, touch the bottom of the well and consequently increase their cell/ECM contact area, but decrease their CDR. In addition, we no longer detect a difference in CDR between small and large wells for inhibitor-treated mESCs.

When mESCs were cultured in small micro-wells they adopt a peculiar cross-shape morphology of their nucleus accompanied with four inwards bend actin arcs along the periphery of the cell. This can be taken as an indicator of a cell with a high membrane tension and a soft nucleus. This cell shape is not only accompanied with a high CDR but also with a high nuclear/cytoplasmic YAP ratio. When the intracellular tension is relieved by applying the inhibitors Blebbistatin or ML-7, the cell shape is not maintained and both CDR and n/c YAP ratio drop. This change in morphology is in line with other reports, where Blebbistatin treatment led to a loss of shape and flattening of cells^56^. A similar situation was already described: Thorpe and coauthors suggested that in case of low nuclear stiffness and high actin contractility the nucleus becomes the major site of mechanotransduction^38^. Indeed, it was also shown that a stretched nucleus favors open chromatin organization and thereby self-renewal of mESCs via altered gene expression, e.g., via OCT4^57,58^. Due to the clearly defined composition of our system it can be excluded that the type of ECM protein, substrate stiffness or the accumulation of soluble factors differentially affects single mESC behavior. Taken together our findings suggest that mechanotransduction induced by the three-dimensional adhesion geometry can positively influence the cell division rate of mESCs.

In summary, our study presents proof-of-principle data on the context-dependent subcellular localization of the mechanotransducer YAP in single mESC. We demonstrate that the adhesion geometry is important in regulating cell cycle progression and n/c YAP ratio in mESCs through intracellular mechanical forces. Further parameters that affect cell fate decisions such as substrate stiffness, geometry or ligand presentation can now easily be monitored in single mESCs. The combination of 2.5D micro-scaffolds with adjustable geometries and a spatially defined bio-functionalization will help to understand the impact of biophysical factors on stem cell behavior.

## Materials and methods

### Microcontact printing

Micropatterned 2D substrates were produced as described elsewhere^59^. In brief, the pattern was transferred from a silicone (Polydimethylsiloxane (PDMS), Sylgard 184, Dow Corning, #(400)000105989377) stamp onto a titanium-gold (Ti: 2 nm, Au: 20 nm thickness) sputtered cover slip. The pattern consisted of octadecylmercaptan (ODM, 1.5 mM in ethanol, Aldrich Chemistry, #01858-25ML). Remaining regions were passivated with HS-C11-(EG)6-OH (0.1 mM in ethanol, Prochimia, #TH-001-m11.n6-0.01). For protein coating, fibronectin (FN, Sigma-Aldrich #F2006) or laminin (LN, Invitrogen, #23017-015) was diluted in PBS and used in a final concentration of 10 µg/ml. Incubation occurred for 30 min at RT. As a control, scaffolds were coated with Poly-d-Lysine (PDL, Gibco, #A38904-01) in a concentration of 50 µg/ml diluted in ddH_2_O for one hour at RT. The proteins adsorb to the ODM-covered regions but not to the HS-C11-(EG)6-OH-covered regions, which allows patterning of the substrate.

### Direct laser writing (DLW) and molding

Plasma cleaned and silanized (1 mM 3-(Trimethoxysilyl)propyl methacrylate, Sigma Aldrich #M6514-25ML in toluene, Carl Roth, #7115.1) cover slips were used for the micro-well fabrication. The master structure for the molding procedure as well as micro-wells with non-adhesive walls were produced via DLW as described elsewhere^37^. In brief, the master structure was fabricated from IP-L (Nanoscribe) photoresist with the following parameters: 25 × objective dipped into the photoresist, 5 cm/s writing speed and 30 mW average laser power. The sample was developed in propylene glycol methyl ether acetate (mr-Dev 600, micro resist technology) for 20 min. and cleaned in isopropanol (Roth, #6752.2). Subsequently the structure was coated with a 100 nm thick layer of Al_2_O_3_ via atomic layer deposition^60^ and silanized with octadecyltrichlorosilane (Sigma, #8.22170). Next, a silicone stamp was produced from this master. After three days of curing at room temperature (RT) the silicone stamp was ready to use. The photoresist Ormocomp (micro resist technology GmbH) was applied onto the silicone stamp and a silanized cover slip was placed on top. Curing occurred for 1 min. under UV light. The structure was developed in methyl isobutyl ketone (Roth, #0338) and isopropanol in a volume ratio of 1:1, rinsed with isopropanol and dried with nitrogen. Before coating, the structures were washed with 0.1% Triton X-100 in PBS. Protein coating was performed as described for micro-islands.

The micro-wells with non-adhesive walls were also fabricated by DLW but the photoresist trimethylolpropane ethoxylate triacrylate (TPE-TA, Sigma, #412171) mixed with 3% (w/w) of the photoinitiator Irgacure 369 (BASF) was used for the walls, with the following parameters: 100 × oil-immersion objective NA = 1.4, 50–200 µm/s writing speed, 10–20 mW laser power. The structures were developed as the molded structures. All five 2D conditions or all five 2.5D conditions, respectively, have been present on the same coverslip to assure equal treatment during all experimental steps, including scaffold fabrication, coating and seeding.

### mESC culture

The mESC line OCT4-eGFP, expressing eGFP under the OCT4-promoter^34^, was kindly provided by Prof. Rolf Kemler (MPI, Freiburg). These experiments were performed according to European (Council Directive 86/609/EEC) and German (Tierschutzgesetz) guidelines for the welfare of experimental animals. mESCs were cultivated at 37 °C, 5% CO_2_ and 95% humidity. The cells were maintained on gelatin (from porcine skin, 0.1%, Sigma, #G6144-100G in PBS) coated flasks on top of a layer of mitotically inactivated mouse embryonic fibroblasts (MEFs). Passaging occurred every 2–3 days. The medium contained DMEM (PAN-Biotech, #P04-03590), 15% Pansera ES (PAN-Biotech, #P30-2602), 1 × non-essential amino acids (Gibco, #M714S), 0.1 mM β-mercaptoethanol (Roth, #4227.1) and penicillin/streptomycin (40 U/ml/40 µg/ml, Sigma, #P-0781). Additionally, cell culture supernatant containing Leukemia inhibitory factor (LIF) was applied in a dilution of 1:50. The LIF secreting COS-7 cells^61^ were cultivated in α-MEM (Gibco, #12561-056) with 10% fetal calf serum (HyClone, #SH30541.03). Passaging occurred every 2–3 days. To separate mESCs from MEFs for experiments, a preplating was performed. The cells were detached and seeded in a plain cell culture flask. MEFs adhered faster than mESCs. After 30–45 min., the mESC containing supernatant was used for experiments with seeding densities between 5.000 and 20.000 cells/cm^2^. The seeding density was chosen to be this low because in this study, we investigated the behavior of single mESCs. Since mESCs were not directly placed on 2D micro-islands or into the 2.5D micro-wells by a micromanipulator, they were seeded as a suspension. The low seeding density reduces the number of cases with two or more cells on 2D-islands or in the 2.5D micro-wells. Inhibitor experiments were performed with 10 µM Blebbistatin (Sigma, #B0560) or 25 µM ML-7 (Sigma, #I2764), to decrease cytoskeletal tension.

### Immunocytochemistry

Samples were fixed after 4 or 24 h with 4% (w/v) paraformaldehyde (Merck, #1040051000). A standard staining procedure was performed with the following antibodies and dyes (final concentration): Rabbit anti-FN (1.2 µg/ml, Sigma, #F3648), rabbit anti-LN (5 µg/ml, Sigma, #L9393), mouse anti-YAP (0.33 µg/ml, Santa Cruz, #sc-101199; applied at 4 °C over night), goat anti-mouse Cy3 (7.5 µg/ml, Jackson ImmunoResearch, #115-165-166), goat anti-rabbit AF568 (7.5 µg/ml, Jackson ImmunoResearch, #111-165-144), goat anti-rabbit AlexaFluor (AF) 647 (7.5 µg/ml, Jackson ImmunoResearch, #111-606-045), DAPI (2–0.2 µg/ml, Roth, #6335.1), Phalloidin AF568 (1 U/ml, Invitrogen, #A12380), Phalloidin AF647 (2 U/ml, Invitrogen, #A22287).

### Image acquisition

The following microscopes and objectives (Carl Zeiss) were used: Axiovert 200 M, EC Plan-Neofluar 10 × /0.3 Ph1, Camera: AxioCam MRm, 60-C 1″ 1.0 × 456,105 by Zeiss; AxioImager.Z1, Acroplan 20 × 0.5w/Ph2, Camera: AxioCam MRm 60 N-C 1″ 1.0 × 426,114 by Zeiss, 10 × Motorized Reflector Changer (HE GFP, HE DsRed, DAPI, Cy5, 47 CFP, Yellow Apotome Calibration, Analy. DIC Trans.Light); LSM510 META, C-Apochromat 40 × /1.2 W Korr or LCI Plan-Neofluar 63 × /1.3 DIC ImmKorr Filters: Cy5/Alexa647 LP650, Cy3/Alexa568 BP575-615, Alexa488 BP505-550, DAPI 420–480, Lasers: 633 nm, 561 nm, 488 nm, 405 nm, Detector: Photomultiplier Tube (PMT); Leo 1530 scanning electron microscope. The software for image acquisition was ZEN (Carl Zeiss). 3D reconstructions were made from confocal image stacks with Imaris (7.7.1, Bitplane). The brightness and contrast of representative images was adjusted with Fiji^62^. The figures were composed with CorelDRAW X7.

### Determining cell division rate (CDR)

To determine the cell division rate (CDR) of single mESCs we imaged the whole field of micro-islands or micro-wells of different sizes with a 10 × dip-in objective four hours after seeding. The positions of single, OCT4-positive mESCs were noted and the culturing was continued until 24 h after seeding, when the experiment was stopped. Although the cell cycle length of mESCs is 10–14 h, we chose a time frame of 24 h, since our culture system, which only provides the basic cues for cell growth, does not represent the optimal growth conditions; the cell cycle might therefore be elongated. In addition, we did neither synchronize the cells nor distinguish between naïve and primed, accordingly the time frame of 24 h compensates for the heterogeneity within the mESC population. After 24 h the whole field of micro-islands or micro-wells of different sizes were imaged again. The positions noted after four hours were located and the number of nuclei on the respective micro-island or in the respective micro-well was counted to determine the number of cells. After 24 h all nuclei were counted, irrespective of their OCT4-expression. If there were two or more nuclei, this was counted as cell division, if there was still one nucleus this was counted as no cell division. For representative images the 40 × high NA objective was used to acquire z-stacks.

### Relative YAP localization

Single mESCs were fixed, stained and imaged 4 h after seeding. To determine the YAP localization, three optical slices from the central part of a z-stack were analyzed. The outline of the cell was determined by an actin staining, the outline of the nucleus by a DAPI staining. Since mESCs have large nucleoli which neither exhibit DAPI nor YAP signals, these areas were excluded from the measurement. The mean fluorescence intensity was measured using Fiji and was normalized to the measured area to obtain the intensity *I*. The relative nuclear to cytoplasmic (n/c) YAP ratio was determined:

$$ \frac{{\text{n}}}{{\text{c}}}{\text{YAP}} = \frac{{{\text{Inucleus}} - {\text{Inucleoli}}}}{{{\text{Icytoplasm}}}}\quad {\text{Icytoplasm}} = {\text{Icell}} - \left( {{\text{Inucleus}}{-}{\text{Inucleoli}}} \right) $$

### Statistical analysis

For all cases a two-tailed student’s t-test was performed. *p* values are indicated in the graphs. *N* represents the number of independent experiments whereas the total number of evaluated cells is indicated as white numbers in the bars. Error bars represent ± one standard deviation.

## Supplementary Information

Supplementary Information 1.

Supplementary Information 2.

Supplementary Information 3.

Supplementary Information 4.

Supplementary Figures.

Supplementary Information 5.

Supplementary Information 6.

Supplementary Information 7.

## Data Availability

All data supporting the finding of this study are included within the paper and its supplementary information files. The datasets generated during the current study are included in the supplementary information.
