# Supplementary material for: Geometrically defined environments direct cell division rate and subcellular YAP localization in single mouse embryonic stem cells
Source: Sci Rep. 2021 Apr 29;11:9269. doi: 10.1038/s41598-021-88336-y (PMC8084931; doi:10.1038/s41598-021-88336-y)
Supplement: Supplementary file 5 — Supplementary Figures. [file 41598_2021_88336_MOESM5_ESM.pdf]

Title

Geometrically defined environments direct cell division rate and subcellular YAP localization in single mouse embryonic stem cells

Authors

Sarah Bertels[1,2,6,ǂ], Mona Jaggy[1,2, ǂ], Benjamin Richter[1], Stephan Keppler[1,6], Kerstin Weber[1], Elisa Genthner[1,6], Andrea C. Fischer[1,3], Michael Thiel[4], Martin Wegener[3,5,6], Alexandra M. Greiner[1], Tatjana J. Autenrieth[1,2], Martin Bastmeyer[1,2,6,7*]

Affiliations

[1] Zoological Institute, Cell- and Neurobiology, Karlsruhe Institute of Technology, Fritz-Haber-Weg 4, 76131 Karlsruhe, Germany

[2] Institute of Functional Interfaces, Karlsruhe Institute of Technology, Hermann-von-Helmholtz-Platz 1, 76344 Eggenstein-Leopoldshafen, Germany

[3] Institute of Applied Physics, Karlsruhe Institute of Technology, Wolfgang-Gaede-Straße 1, 76131 Karlsruhe, Germany

[4] Nanoscribe GmbH, Hermann-von-Helmholtz-Platz 1, 76344 Eggenstein-Leopoldshafen, Germany

[5] Institute of Nanotechnology, Karlsruhe Institute of Technology, Hermann-von-Helmholtz-Platz 1, 76344 Eggenstein-Leopoldshafen, Germany

[6] 3DMM2O – Cluster of Excellence (EXC-2082/1 – 390761711)

[7] Institute of Biological and Chemical Systems – Biological Information Processing, Karlsruhe Institute of Technology, Hermann-von-Helmholtz-Platz 1, 76344 Eggenstein-Leopoldshafen, Germany

[ǂ] Co-first authors

* Corresponding author: Martin Bastmeyer, Zoological Institute, Cell- and Neurobiology, Karlsruhe Institute of Technology, Fritz-Haber-Weg 4, 76131 Karlsruhe, Germany, Phone: +49 721 608-4-3085, Email: bastmeyer@kit.edu

Supplementary Information

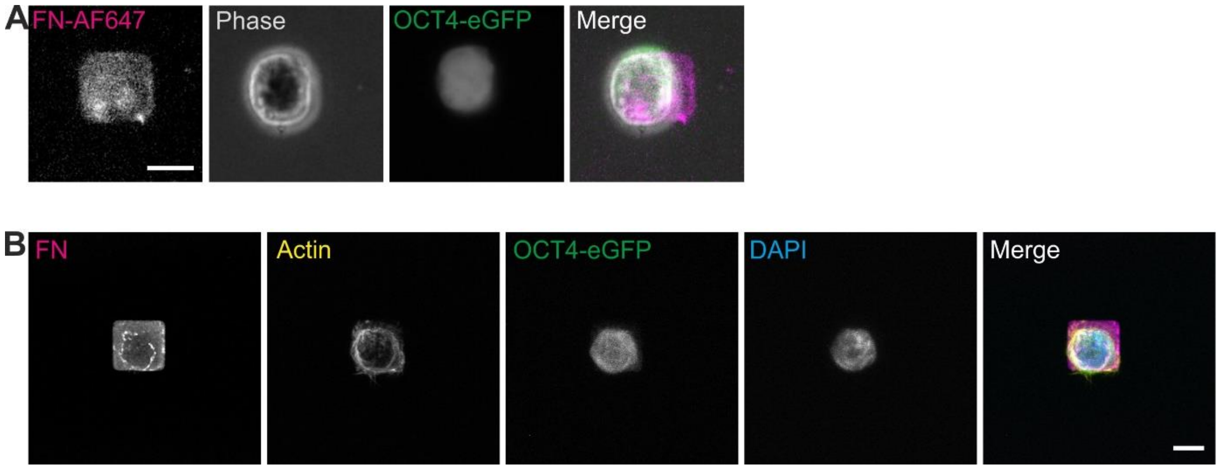

Supplementary Figure 1: Small micro-islands have the size of single mESCs

(A) Live cell imaging four hours after seeding shows an OCT4-eGFP positive single mESC on a (15 × 15) µm^2 micro-island. The micro-island is visualized by AlexaFluor647-labeled Fibronectin (magenta). The Phase contrast (grey) image and the nucleus (marked by OCT4-eGFP) show that the mESC covers the largest part of the small micro-island. (B) Fluorescence image of a single mESC, fixed and immunolabeled after 48 hours of incubation. The actin (yellow) labeling shows that the cell fills the micro-island completely. The cells still show OCT4-eGFP expression. Scale bars: 10 µm

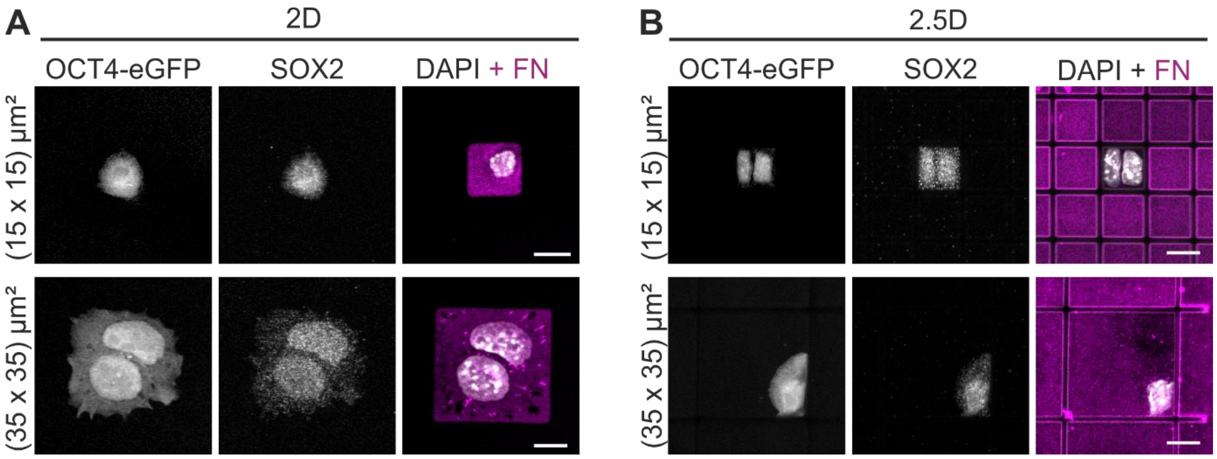

Supplementary Figure 2: Pluripotency marker expression in mESCs

The two pluripotency markers OCT4 and SOX2 are expressed in mESCs cultured on (A) 2D micro-islands and in (B) 2.5D micro-wells for 24 hours. The nucleus is depicted in white and fibronectin in magenta. Scale bars: 10 µm

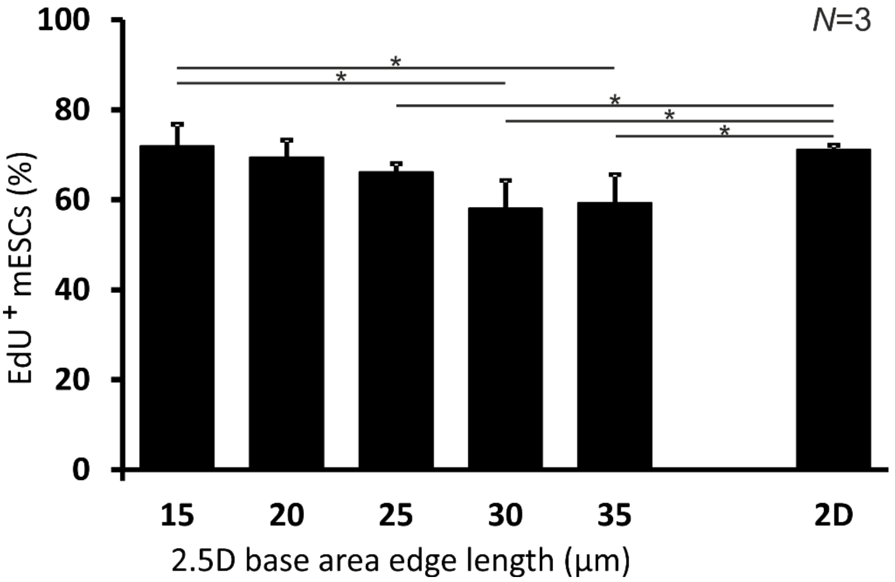

Supplementary Figure 3: Incorporation of EdU in dividing mESCs

The mESCs were seeded into fibronectin-coated 2.5D scaffolds of different size and incubated with EdU. The EdU-positive cells were counted 4 hours after seeding to determine the percentage of cells in S-phase. In the smallest micro-wells the percentage of EdU^{+} cells is significantly higher than in large micro-wells. Strikingly, the percentage of EdU^{+} mESCs in small micro-wells does not significantly differ from the percentage of EdU^{+} mESCs on a homogeneously coated 2D surface. Graphs show mean ± one s.d. from N=3 independent experiments. Asterisk indicates a p-value lower than 0.05 that was determined by a two-tailed student's t-test.

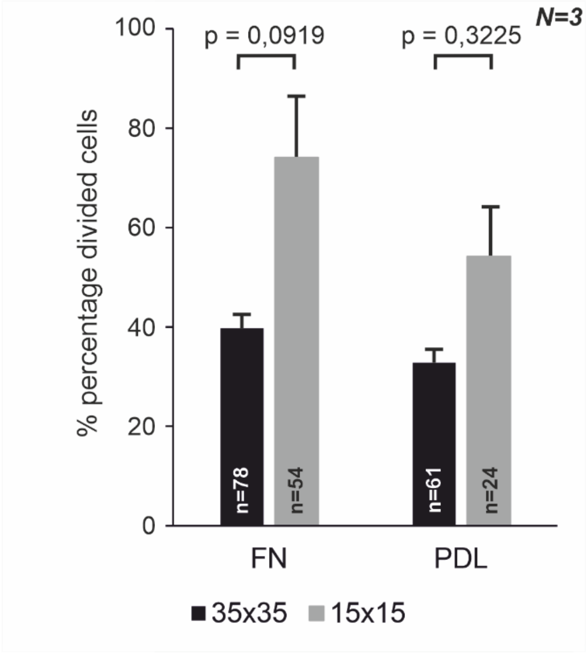

Supplementary Figure 4: Division rate in PDL-coated 2.5D scaffolds

The mESCs were seeded into fibronectin- or PDL-coated 2.5D scaffolds. CDR on FN decreases with increasing micro-well size and reveals a similar trend in PDL-coated scaffolds. Graphs show mean of all n ± one s.d. from *N*=3 independent experiments. p-values were determined by a two-tailed student's t-test.

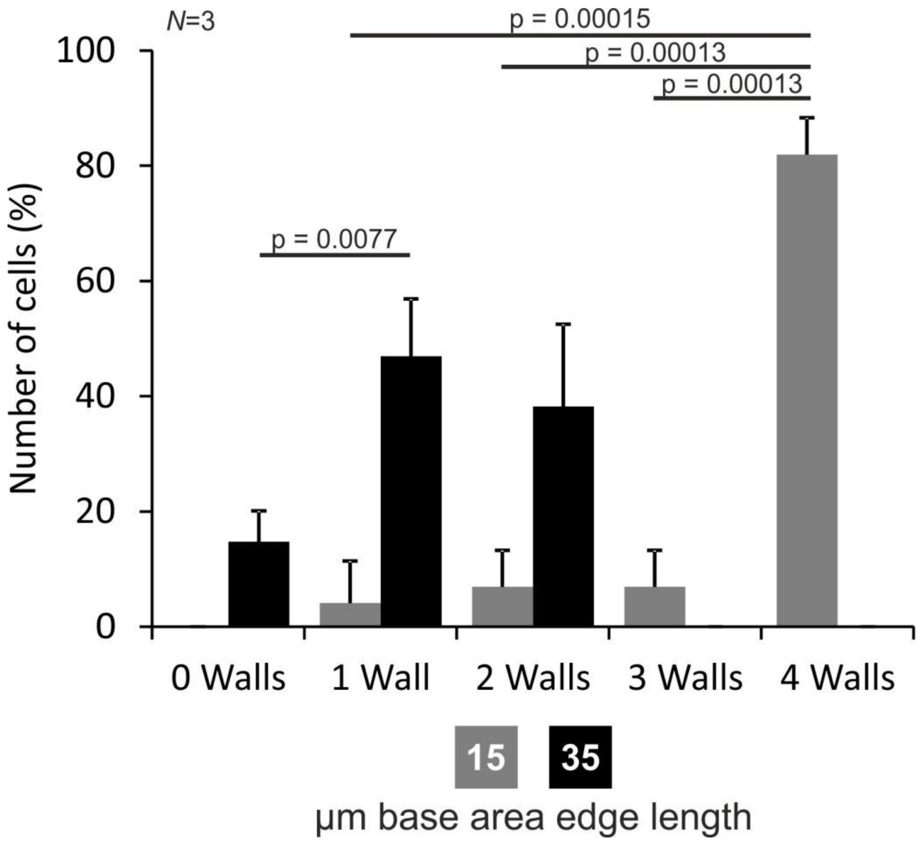

Supplementary Figure 5: Adhesion of mESCs to walls of 2.5D micro-scaffolds

Four hours after seeding, ~ 80% of mESCs cultured in small micro-wells (15 µm base area edge length) contacted all four walls. In contrast, in large micro-wells (35 µm base area edge length) ~ 85% of mESCs adhered to either one or two walls, whereas the rest remained in the middle. Graphs show mean ± one s.d. from *N*=3 independent experiments. p-values were determined by a two-tailed student's t-test.

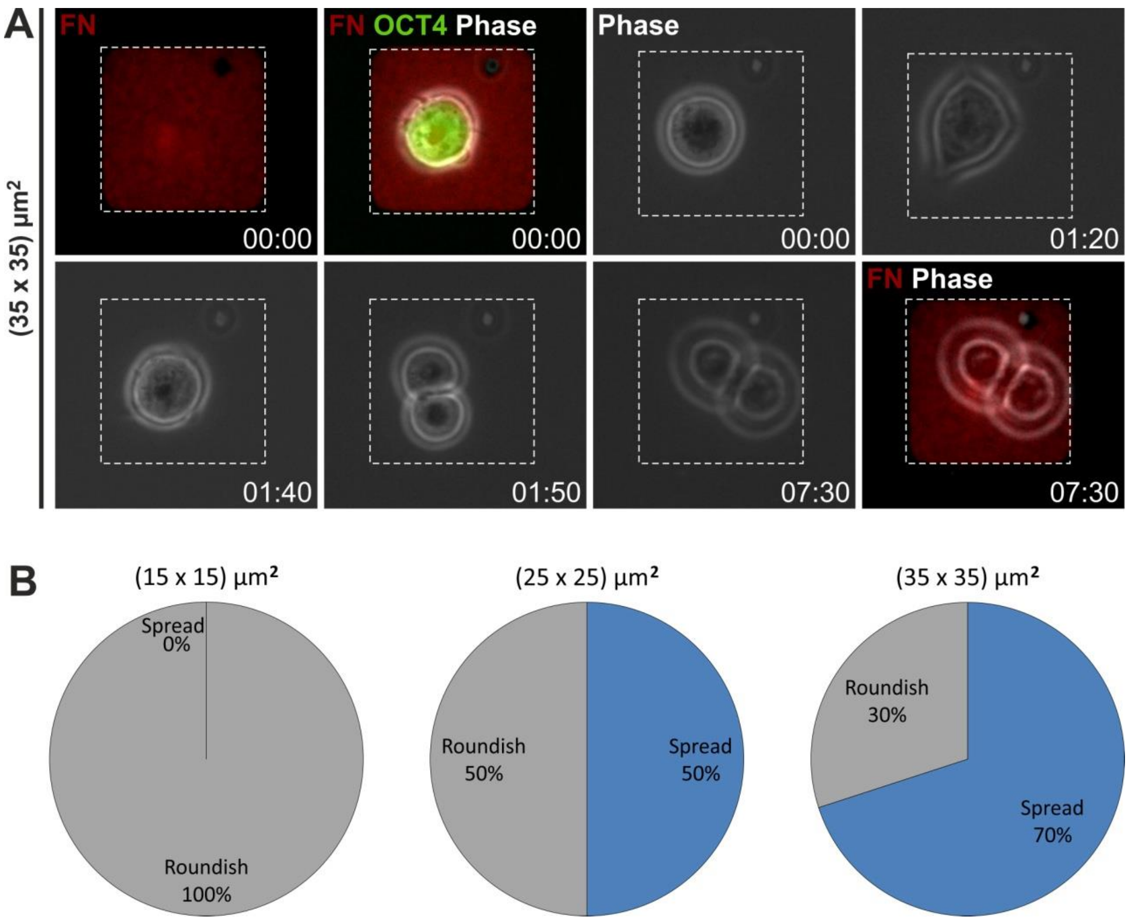

Supplementary Figure 6: Live cell imaging of single OCT4-eGFP positive mESCs on 2D micro-islands

(A) Representative time-lapse images showing the behavior of a single OCT4-eGFP positive mESC (green) on a large FN-coated micro-island ((35 × 35) μm^2, red). The initially round mESC adheres to the micro-island, spreads, and then rounds up before cell division. To visualize the micro-islands during time-lapse microscopy FN was labeled with AlexaFluor647. Phase contrast images were taken every 10 min. (B) Quantification of mESC morphology on 2D micro-islands (15, 25, 35 µm base area edge length). Cells from six experiments were investigated for 17 hours by time-lapse microscopy. It was documented whether mESCs spread before they undergo cell division. We found that mESCs on small micro-islands stay round while mESCs on larger micro-islands spread. On the largest micro-islands 70% of the mESCs spread before they round up for cell division.
